# Human NK Cell Development: One Road or Many?

**DOI:** 10.3389/fimmu.2019.02078

**Published:** 2019-08-29

**Authors:** Frank Cichocki, Bartosz Grzywacz, Jeffrey S. Miller

**Affiliations:** ^1^Department of Medicine, University of Minnesota, Minneapolis, MN, United States; ^2^Department of Laboratory Medicine and Pathology, University of Minnesota, Minneapolis, MN, United States

**Keywords:** NK cell, development, precursor, innate, adaptive, progenitor, immune, differentiation

## Abstract

CD3^−^CD56^+^ NK cells develop from CD34^+^ hematopoietic progenitors (HPCs) *in vivo*, and this process can be recapitulated *in vitro*. The prevailing model is that human NK cell development occurs along a continuum whereby common lymphocyte progenitors (CLPs) gradually downregulate CD34 and upregulate CD56. Acquisition of CD94 marks commitment to the CD56^bright^ stage, and CD56^bright^ NK cells subsequently differentiate into CD56^dim^ NK cells that upregulate CD16 and killer immunoglobulin-like receptors (KIR). Support for this linear model comes from analyses of cell populations in secondary lymphoid tissues and *in vitro* studies of NK cell development from HPCs. However, several lines of evidence challenge this linear model and suggest a more branched model whereby different precursor populations may independently develop into distinct subsets of mature NK cells. A more definitive understanding of human NK cell development is needed to inform *in vitro* differentiation strategies designed to generate NK cells for immunotherapy. In this review, we summarize current evidence supporting the linear and branched models of human NK cell development and the challenges associated with reaching definitive conclusions.

## The Plasticity of Early Human Hematopoiesis

The population of cells comprising human blood is organized as a cellular hierarchy derived from multipotent stem cells. The first *in vivo* experiments demonstrating reconstitution of the hematopoietic system from stem cells were based on rescue of lethal irradiation by bone marrow transplant in mice (1). Subsequent bone marrow transplant experiments in mice provided estimates of the minimal number of hematopoietic stem cells (HSCs) that could reconstitute hematopoiesis (2) and revealed *in vivo* proof for the multipotent nature of stem cells (3). The advent of flow cytometry and cell sorting allowed for purification of hematopoietic stem cells and demonstration that a small number of these cells could reconstitute all blood cell types in lethally irradiated mice (4).

Throughout the past two decades there have been numerous studies characterizing hematopoietic stem cells and determinants of self-renewal or differentiation. In early models of the hematopoietic differentiation tree, the first branch point segregated common lymphoid progenitor cells (CLPs) from common myeloid progenitors (CMPs). Subsequent modifications to the tree have been made based on work showing that the HSC pool is very heterogeneous in terms of self-renewal and differentiation properties. One landmark discovery that challenged the standard branched tree paradigm of human hematopoiesis was the identification of a population of multi-lymphoid progenitor cells (MLPs) that could generate all lymphoid cell types, as well as monocytes, macrophages, and dendritic cells (DCs). MLPs were characterized as a distinct Thy-1^neg−low^CD45RA^+^ population within the CD34^+^CD38^−^ HSC pool of both cord blood and bone marrow. When cultured on the MS-5 murine stromal cell line, MLPs differentiated into myeloid cells, B cells, and NK cells at a nearly 1:1:1 ratio. A large fraction of MLPs could also differentiate into T cells when cultured on OP9 murine stromal cells transduced with the Notch ligand DL1 (5). This work, along with other studies showing macrophage potential in thymic progenitors, CLPs, and B cell progenitors call into question the lymphoid-restricted state of the presumed CLP (6–10) and led to a model whereby multipotential progenitors (MPPs) initially differentiate into lymphoid-primed multipotential progenitors (LMPP) (11–14) in route to definitive myeloid and lymphoid commitment (15, 16).

Several important conclusions can be drawn from these studies. First, there exists considerable heterogeneity and plasticity with regards to hematopoiesis and lineage potential of precursors. Second, precursors with some degree of B and T cell lineage restriction appear to retain NK cell and myeloid potential. From an evolutionary perspective, the innate myeloid and NK cell lineage pathways may represent ancestral programs that are retained in progenitors. Adaptive immunity, when it arose, may have been layered onto the ancestral programs, resulting in further hematopoietic lineage diversification. Third, signals within the microenvironment in which a progenitor resides provide instructive signals that strongly influence the developmental path of a given progenitor.

## NK Cell Precursors and Ontogeny

One of the first reports aimed at defining the precursor origin of NK cells was performed by Kumar and colleagues in the mid 1980's. The authors transplanted syngeneic bone marrow cells into lethally irradiated mice that were also depleted of NK cells by injection of an anti-asialo GM1 antibody. Using this system, the authors demonstrated that an intact bone marrow microenvironment was necessary for the development of mature, lytic NK cells, and that NK cell precursors lack expression of several surface antigens that define mature NK cells (17). Subsequently, an early foray into human NK cell ontogeny was undertaken by Lanier et al. who characterized freshly isolated NK cells from fetal tissue. The most striking finding from this study was that fetal NK cells, in contrast to adult peripheral blood NK cells, expressed intracellular (but not surface) CD3δ and CD3ε. This led to the hypothesis that NK cells and T cells may share a common precursor that splits to the T or NK cell lineage depending on environmental cues (18). Contemporaneously, Reinherz and colleagues identified a dominant fetal thymocyte population in mice lacking expression of CD4 and CD8 but expressing Fc gamma RII/III prior to TCR acquisition *in vivo*. If maintained in a thymic environment, these precursors exhibited stepwise differentiation into canonical CD8^+^ T cells. If removed from the thymus, these precursors developed into canonical NK cells with cytotoxic function (19). Subsequently, *in vitro* fetal thymic organ culture experiments using mouse fetal thymocytes demonstrated that a T/NK-committed progenitor defined as NK1.1^+^CD117^+^CD44^+^CD25^−^ could efficiently develop into T cells if cultured in a thymic microenvironment, whereas co-culture with bone marrow-derived stromal cells resulted in the generation of mature NK cells (20). Support for a developmental relationship between NK cells and T cells also comes from whole-genome microarray analyses of murine splenic leukocyte populations. At the transcriptome level, NK cells and T cells cluster within a complex that is distinct from those formed by subsets of B cells, DCs, and macrophages by principal components analysis (21).

Compelling evidence exists for the idea that T cell-determining factors are needed to enforce the development of precursor cells into the T cell lineage, and the NK cell lineage becomes the default pathway in the absence of these factors. Several murine studies have shown that one of the earliest checkpoints in T cell development is dependent on the zinc-finger transcription factor Bcl11b. Bcl11b-deficient mice exhibit impaired thymocyte development between the DN3 to immature SP stage because of an inability to rearrange the TCR V_β_ to D_β_ gene segments (22). Genetic deletion of Bcl11b in conditional knockout mice results in a loss of T cell identity in developing DN3 thymocytes and reprograming to a morphological and transcriptional state resembling that of NK cells (23–25). One interpretation of these results is that early progenitor cells with intrinsic T cell potential but low or absent Bcl11b expression differentiate into NK cells, providing support for the existence of a common T/NK progenitor. Another interpretation is that Bcl11b expression is necessary to enforce T cell identity during development by overriding a more ancestral NK-like program, and there is no actual NK/T lineage split determined by Bcl11b. The latter interpretation seems more likely based on a report of a patient that contained a mutant *BCL11B* variant causing dysregulated binding of BCL11B to promoter targets. The patient exhibited a “leaky” form of severe combined immunodeficiency (SCID) and very low T cell counts. However, NK cell counts were within the normal range (26).

There has been a continuous evolution regarding our understanding of the earliest stages of progenitor cell commitment to the NK cell lineage. An updated model of human lymphopoiesis has been put forth postulating that lymphoid development stems from distinct populations of CD127^−^ and CD127^+^ early lymphoid progenitors (ELPs). Evidence for this model is supported by experiments where CD34^+^ HSCs were engrafted into immunodeficient mice and subsequently phenotyped for surface expression of various lineage markers. Representation of flow cytometry data using tree-plots suggested that lymphoid cells differentiated along two pathways, distinguished by expression of CD127, that originates from CD34^high^CD45RA^+^ progenitors. A series of *in vitro* differentiation assays showed that CD127^−^ ELPs could generate T cells, marginal zone B cells, NK cells, and innate lymphoid cells (ILCs), while CD127^+^ ELPs could generate marginal zone B cells, NK cells, and ILCs. Molecular characterization of *in vitro*-generated NK cells identified substantial differences according to whether cells originated from CD127^−^ or CD127^+^ NKIPs. NK cells derived from CD127^−^ NKIPs expressed higher levels of *GZMB, IFNG*, and *GZMK* and secreted higher levels of IFN-γ and TNF-α when stimulated with PMA. NK cells derived from CD127^+^ NKIPs expressed higher levels of genes encoding several transcription factors including *RUNX1, TCF4, NFIL3, MYC, LEF1, EOMES, ETS1, TCF12*, and *BCL11B* and exhibited marginally higher degranulation in response to K562 stimulation (27). Further dissection of the relative contribution of CD127^−^ and CD127^+^ NKIPs to the mature peripheral blood NK cell pool and NK cell subsets in various tissues will be of interest.

Because of the complexity and plasticity of early hematopoiesis and lineage commitment, it has been challenging to define lineage-restricted NK cell progenitors. A foundational study by Chen and colleagues showed that a subpopulation of CD34^+^Lin^−^CD45RA^+^ cells expressing CD10 could give rise to T cells, B cells, NK cells, and DCs under supportive culture conditions (28). Similar results were reported by Miller et al. who demonstrated that 2% of bone marrow cells with a CD34^+^Lin^−^CD38^−^ phenotype could give rise to at least three lineages (NK cells, B cells, and myeloid cells) under the same culture conditions (29). Subsequent work comparing lymphoid potential of CD34^+^Lin^−^CD45RA^+^ cells isolated from cord blood concluded that CD34^+^Lin^−^CD45RA^+^CD10^+^ progenitors predominantly exhibited B cell potential, while CD34^+^Lin^−^CD45RA^+^CD7^+^ progenitors skewed more toward the T cell and NK cell lineages when differentiated *in vitro* (30). Support for CD7 expression by the putative NK cell progenitor came from experiments showing a high cloning efficiency of CD3^−^CD56^+^ NK cells from CD34^+^CD7^bright^ bone marrow progenitors (31). Another study of lymphoid and myeloid lineage commitment using precursors from cord blood described B cell and NK cell potential from CD34^+^CD38^−^CD10^+^CD7^+^ progenitors with barely detectable expansion of these cells in myeloid stromal cultures (32). An important step forward in identifying a lineage-restricted NK cell progenitor was made about a decade later with the identification of a very rare population of cord blood and bone marrow progenitors with a Lin^−^CD34^+^CD38^+^CD123^−^CD45RA^+^CD7^+^CD10^+^CD127^−^ phenotype that gave rise exclusively to NK cells when co-cultured at limiting dilutions with supporting stroma and after transplantation into newborn immune-deficient mice. These NK cell precursors were shown to be “downstream” of CLP-like cells with a Lin^−^CD34^+^CD38^+^CD123^−^CD45RA^+^CD7^+^CD10^+^CD127^+^ phenotype (33). It would be of considerable interest to revisit the place of these lineage-restricted NK cell precursors in the hematopoietic hierarchy in the context of the “two-family” model, which posits that CD127^−^ and CD127^+^ ELPs differentiate independently and can each give rise to NK cells (27).

Another population of lineage-restricted progenitor cells with NK and T cell potential characterized as Lin^−^CD34^+^DNAM-1^bright^CXCR4^+^ has been described. These cells were bone-marrow-resident, but increased markedly in circulation in individuals with chronic infections. *Ex vivo* culture of these cells with cytokines (FLT3, SCF, IL-7, IL-15) led to the development of NK cells and TCRα/β^+^ T cells, but not myeloid cells. In contrast, cord blood-derived CD34^+^DNAM^−^CXCR4^−^ progenitor cells in the same culture conditions gave rise to NK and myeloid cells (34). Where these Lin^−^CD34^+^DNAM-1^bright^CXCR4^+^ progenitor cells fit within the developmental hierarchy of NK cells is unclear. It will be of interest to determine whether cytotoxic lymphocytes that arise from these unique progenitors play an important role in the anti-viral immune response.

## The Linear Model of NK Cell Development

CD3^−^CD56^+^ NK cells with cytotoxic function can be generated *in vitro* after long-term culture of CD34^+^ cells isolated from cord blood, bone marrow, fetal liver, thymus, or secondary lymphoid tissue with IL-2 or IL-15 (31, 35–38). Based on the anatomical locations of progenitors and their capacity to develop into NK cells under supportive conditions, a stepwise model for development and maturation of human NK cells has been put forth by Freud and Caliguiri. In this model, HSCs give rise to “Stage 1” progenitors that retain CD34 expression and acquire CD45RA and CD10. These cells give rise to “Stage 2” progenitors marked by loss of CD10 expression and acquisition of CD117. “Stage 3” is marked by downregulation of CD34 and acquisition of LFA-1. These cells are presumed to be restricted to the NK cell lineage given their inability to differentiate into T cells or DCs *in vitro* and their capacity for efficient differentiation into bona fide NK cells in response to IL-15. “Stage 4” is marked by acquisition of CD94, and these cells represent the CD56^bright^ NK cell subset. The precursor population for CD56^bright^ cells has been identified as exhibiting a CD34^dim^CD45RA^+^integrin α_4_β_7_ phenotype (38). Further differentiation into “Stage 5” cells is marked by downregulation of CD94 and acquisition of CD16 and killer immunoglobulin-like receptors (KIR). These cells represent the CD56^dim^ NK cell subset (39).

The presumed developmental transition from a CD56^bright^ to a CD56^dim^ phenotype is perhaps the most controversial step in this developmental model. A good case can be made for this developmental pathway. CD56^bright^ NK cells are the predominant population early after hematopoietic cell transplant. Their frequency decreases by 3 months post-transplant, concomitant with an increase in the percentage of CD56^dim^ NK cells (40). While this pattern of NK cell reconstitution could reflect a developmental relationship, an alternative hypothesis is that the abundance of CD56^bright^ NK cells early post-transplant is due to high levels of homeostatic expansion of this subset in the setting of lymphopenia induced by transplant conditioning. Additional support for a developmental relationship between CD56^bright^ and CD56^dim^ NK cells comes from the identification of a functionally and phenotypically intermediate population of CD56^dim^CD94^high^ NK cells that have been described as a transitional population between CD56^bright^ and CD56^dim^CD94^low^ NK cells (41). However, whether human NK cells differentiate from CD56^bright^ to CD56^dim^CD94^high^ to CD56^dim^CD94^low^ has not been definitively established. Interestingly, CD94 has also been used as a marker to define phenotypically and functionally distinct NK cell subsets in mice. Murine CD94^high^ NK cells share phenotypic and functional properties with human CD56^dim^CD94^high^ NK cells. When CD94^high^ and CD94^low^ NK cells were purified and adoptively transferred into congenic mice, CD94^low^ NK cells became CD94^high^ but not vice versa (42).

Perhaps the strongest evidence in support of the idea that CD56^bright^ NK cells differentiate into CD56^dim^ NK cells comes from studies where CD56^bright^ NK cells were sorted and stimulated *in vitro*. In a study by Chan et al., a fraction of sorted CD56^bright^ NK cells co-cultured with synovial fibroblasts exhibited CD56 downregulation and had a phenotype consistent with CD56^dim^ NK cells. The apparent differentiation of CD56^bright^ NK cells to CD56^dim^ NK cells could be inhibited by the addition of an antibody that blocks fibroblast growth factor receptor 1 (FGFR1). Of note, stimulation of CD56^bright^ NK cells with a combination of IL-2, IL-15, and 721.221 cells did not induce differentiation to a CD56^dim^ phenotype. CD56^bright^ NK cells that were adoptively transferred into NOD-SCID mice were almost uniformly CD56^dim^CD16^+^ when analyzed 10 days later in the blood, spleen, and lymph nodes. Furthermore, CD56^bright^ NK cells were shown to have longer telomere repeat lengths relative to CD56^dim^ NK cells, suggesting that they are more naïve (43). A contemporaneous study by Romagnani et al. reported on the acquisition of CD56^dim^ NK cell features such as KIR and CD16 upregulation after stimulation of sorted CD56^bright^ NK cells with IL-2 or IL-15 and confirmed the existence of longer telomere repeats in CD56^bright^ NK cells (44). The discrepancies between these two studies with respect to the role of cytokines in driving maturation of CD56^bright^ NK cells may be due to experimental techniques, but additional studies are needed to gain a more definitive understanding of this stage of NK cell maturation. Indeed, it's somewhat surprising that no follow up studies looking deeper into the FGF signaling pathway and its role in driving NK cell maturation have been published.

The CD56^dim^ NK cell subset in peripheral blood is heterogeneous mix of cells with respect to the expression of KIR, CD94, NKG2A, CD62L, and CD57. Relative surface expression levels of these molecules are indicative of maturation status. The current model based on analysis of peripheral blood NK cells from healthy donors and NK cell reconstitution after hematopoietic cell transplantation suggests that as CD56^dim^ NK cell mature, they downregulate NKG2A and CD62L and subsequently acquire KIR and CD57. Sequential maturation is associated with a gradual decline in proliferative capacity in response to IL-2 or IL-15 (45, 46). The acquisition of inhibitory KIR and NKG2A after lineage commitment has been studied using *in vitro* models of human NK cell development from CD34^+^ precursors (47, 48). However, late stage NK cell differentiation and maturation is difficult to study using current culture conditions. NK cell development from CD34 precursors *in vitro* is a slow process that takes ~4 weeks, and CD3^−^CD56^+^ NK cells generally exhibit low-to-absent expression of KIR, CD16, and CD57. Additionally, signaling through the common γ-chain cytokines IL-2 and IL-15 drives high expression of CD56, NKG2A, and cytotoxic granule components in cultured NK cells. Thus, innovative new approaches need to be developed in order to study the paths of late stage NK cell maturation and the mechanisms that influence NK cell heterogeneity.

## Evidence for a Non-Linear Model of NK Cell Development

The linear model of human NK cell development is a useful construction. Within it lie some fundamental truths, such as the concept that multipotent progenitor cells become lineage restricted and further mature. However, we may need to go beyond this model to understand NK cell heterogeneity. NK cells were once thought to be a relatively homogenous lymphocyte population, particularly in comparison to T and B cells that can generate remarkable receptor diversity through somatic DNA recombination. This view has changed with the advent of more sophisticated technologies for cellular analysis and computing power. Using mass cytometry with a panel of 28 NK cell receptors, Horowitz et al. phenotyped peripheral blood NK cells from five sets of monozygotic twins and 12 unrelated donors with defined KIR and HLA genotypes. Using a Boolean gating strategy to analyze the mass cytometry data, they estimated 6,000–30,000 phenotypic populations within an individual and more than 100,000 phenotypes in the entire donor panel. Interestingly, no single phenotype accounted for more than 7% of the total NK cells, and subsets comprising the top 50 phenotypes accounted for an average of only 15% of a given individual's NK cells. Hierarchical clustering of NK cell populations on the basis of surface receptors showed that the major distinguishing receptors were CD94, NKG2A, CD16, and CD57. Two separate clusters emerged: a less mature CD94^+^NKG2A^+^ cluster and a mature CD16^+^CD57^+^ cluster (49). With the existence of these new technologies and sophisticated methods of analysis, it will be exciting to find out how population frequencies shift in the context of aging and disease. It will also be of interest to know whether less mature populations such as the CD94^+^NKG2A^+^ population continually mature and alter their phenotype or whether they are more static and fixed at their stage of differentiation. It is currently unknown whether the astounding diversity found within the peripheral blood NK cell population is largely a reflection of a spectrum of maturational states and stochastic receptor expression influenced by the environment or whether clonal diversity within the precursor pool dictates NK cell phenotypes. In this section we review evidence for the hypothesis that NK cell diversity could be determined at the precursor level.

The idea that NK cells develop exclusively from CLPs was challenged by experiments showing that CMPs and granulocytic-monocytic precursors (GMPs) isolated from cord blood could efficiently differentiate into NK cells when cultured in the presence of NK-supporting cytokines and stroma. Additionally, NK cells derived from myeloid precursors variably expressed colony-stimulating factor receptor (CSFR) during culture. Both CSFR^−^ and CSFR^+^ progenitors gave rise to functional CD56^+^ NK cells if cultured in NK-supporting conditions, and addition of colony-stimulating factor (CSF) to NK cell cultures skewed development toward the monocyte lineage in a dose-dependent manner. Interestingly, NK cells derived from CSFR^+^ myeloid precursors exhibited significantly higher killer immunoglobulin-like receptor (KIR) expression (50). More KIR acquisition on NK cells derived from myeloid precursors could be related to CSFR, which signals through the transcription factor Myc (51). Upstream distal *KIR* promoters have binding sites for Myc, and Myc overexpression drives *KIR* gene transcription (52). Importantly, a fraction of NK cells with a more mature NKG2A^−^KIR^+^ phenotype was identified in cultures where NK cells were derived from CSFR^+^ progenitors, and this population was absent in cultures where NK cells were derived from CSFR^−^ progenitors (50). Supporting evidence for human NK cell differentiation from myeloid progenitors was reported in a more recent study of NK cell reconstitution in humanized mice. In this model, 80% of CD56^+^ cells in the bone marrow co-expressed myeloid markers such as CD33 or CD36. These cells lacked expression of conventional NK cell markers including NKG2D and NKp46 and were hypofunctional with regards to IFN-γ production and cytotoxicity. However, CD56^+^CD36^+^ NK cells sorted from the bone marrow of these mice and cultured in differentiation media containing stem cell factor (SCF), IL-15, and FLT-3 ligand exhibited maturation toward the conventional NK cell lineage as evidenced by loss of CD36 expression and acquisition of NKp46 and NKG2D. Similar observations were reported using CD56^+^CD36^+^ cells isolated from human cord blood. Finally, the authors demonstrated that when purified CD4^+^CD38^+^CD123^low^CD45RA^+^ cells with a GMP phenotype were cultured in conditions supporting NK cell development, transient CD36 expression was observed followed by significant upregulation of CD56 (53).

It could be argued that NK cell development from myeloid progenitors is an artifact of the culture systems used and that it does not occur *in vivo*. Indeed, further studies need to be done *in vivo* to substantiate *in vitro* results. Nonetheless, given the plasticity of hematopoiesis described above, we believe that it's likely that some fraction of lineage-committed NK cells in humans derive from myeloid precursors. This notion is supported by other studies showing that under certain circumstances NK cells can share properties with DCs, such as MHC class II upregulation and antigen-presentation (54, 55). Conversely, there are conditions under which DCs acquire cytotoxicity characteristic of NK cells (56).

While much of the above discussion has highlighted hematopoietic plasticity and the multi-lineage potential of progenitor cells, results from a recent study by Dunbar and colleagues utilizing autologous transplantation of rhesus macaques with barcode-labeled CD34^+^ cells suggest that the NK cell lineage is ontologically distinct. This contention was based on analysis of peripheral blood from macaques between 3- and 6.5-months post-transplant. Within this window, many shared clones were contributing to the granulocyte, monocyte, T cell and B cell lineages, while the clonal composition of NK cells was distinct. Additionally, distinct clonal patterns were observed for the more abundant CD16^+^CD56^−^ NK cell subset compared to the less abundant CD16^−^CD56^+^ NK cell subset (57).

In a follow up study, the same group reported on NK cell reconstitution from the same rhesus macaques out to 4 years post-transplant. In this subsequent analysis, the differences in clonal contributions to the CD16^+^CD56^−^ and CD16^−^CD56^+^ NK cell populations were still evident, and the CD56^−^CD16^+^ NK cell subset exhibited low clonal diversity. Despite technical challenges related to limited reagents to phenotype macaque NK cells, the authors also showed that reconstituted NK cells segregated by expression of KIR also exhibited clonal segregation. Furthermore, these clonal patterns were maintained after short term *in vivo* depletion with an anti-CD16 antibody. This finding suggests persistence and self-renewal of oligoclonal NK cell populations (58). If it can be assumed that (a) reconstitution of hematopoiesis after adoptive transfer of transduced CD34^+^ progenitors accurately recapitulates NK cell ontogeny, (b) macaque and human NK cell development are reasonably equivalent, and (c) macaque CD16^+^CD56^−^ and CD16^−^CD56^+^ NK cells are analogous to CD56^dim^ and CD56^bright^ NK cells, the results from this study suggest that CD56^bright^ NK cells and CD56^dim^ NK cells are distinct lineages. This has obvious implications for the current model of human NK cell development where CD56^bright^ NK cells are assumed to be precursors of CD56^dim^ NK cells. While the debate over whether CD56^bright^ NK cells are precursors of CD56^dim^ NK cells or an independent lineage may seem somewhat trivial, it has important implications for generating NK cells for immunotherapy. It is possible that current culture systems which predominantly generate cells with a CD56^bright^ phenotype favor the expansion/differentiation of a particular subset of precursor clones at the expense of other clones that differentiate into CD56^dim^ NK cells.

## Considerations of NK Cell Development in Relation to ILCs

In recent years, much knowledge has been gained by studying NK cell development in parallel with the closely related ILCs and lymphoid tissue inducers (LTi). In one report describing committed ILC precursors, the immune systems of PLZF^GFPcre+/−^ mice carrying the ROSA26-floxstop-yellow fluorescent protein fate (YFP)-mapping allele were analyzed in detail. GFP marked cells actively expressing promyelocytic leukemia zinc finger (PLZF), and YFP marked cells that had previously expressed PLZF at some point during their development. PLZF is a transcription factor that plays an important role in the effector differentiation of NKT cells (59, 60). Hematopoietic reconstitution experiments using progenitor cells from PLZF^GFPcre+/−^ mice demonstrated that the vast majority of NKT cells expressed YFP, whereas CLPs, B cells, and T cells were unlabeled. ILC1, ILC2, and ILC3 cells were YFP-labeled to varying extents. Interestingly, non-recirculating DX5^−^CD49a^+^CD3ε^−^NK1.1^+^ NK cells in the liver were heavily labeled, whereas classical recirculating DX5^+^CD49a^−^ NK cells were mostly negative. In a search for the PLZF-expressing ILC precursor, the authors identified a rare subset of PLZF^high^ cells in fetal liver and adult bone marrow with a Lin^−^IL-7Rα^+^cKit^+^α4β7^high^ phenotype that demonstrated ILC1, ILC2, and ILC3 potential at the clonal level. This potential excluded classical LTi and NK cells, but included non-recirculating DX5^−^CD49a^+^CD3ε^−^NK1.1^+^ NK cells. The results of this study suggest that liver-resident NK cells share a common progenitor with ILCs and that a distinct PLZF^−^ progenitor gives rise to circulating NK cells (61). Whether PLZF expression is associated with the divergence of canonical NK cells and ILCs in humans has not yet been determined, and there may be important differences in PLZF expression patterns between species that limit the application of knowledge gained from these mouse experiments to human biology. In contrast to mice, recirculating canonical NK cells in humans are PLZF^+^, and PLZF downregulation through promoter DNA methylation is a hallmark of adaptive NK cells that arise in response to human cytomegalovirus (HCMV) (62).

Mice with an inhibitor of DNA binding 2 (Id2) reporter allele (*Id2*^Gfp/+^) have also been employed to track ILC progenitors (63). Id2 is a transcriptional regulator and inhibitor of E proteins (64). Genetic deletion of *Id2* in mice abrogates the development of all ILC lineages, including NK cells (65, 66). In *Id2*^Gfp/+^-reporter mice, a Lin^−^Id2^+^IL-7Rα^+^CD25^−^${\text{α}}_{4}{\text{β}}_{7}^{+}$ cell population representing a common progenitor to the ILC1, ILC2, and ILC3 lineages was identified. This progenitor population was termed the common progenitor to all helper-like ILCs (CHILP). CHILP cells did not give rise to conventional NK cells in adoptive transfer experiments, indicating early divergence of the ILC and NK cell lineages (63). However, this interpretation has been recently challenged by DiSanto and colleagues who studied ILC and NK cell development using Id2^RFP^-reporter mice. The genomes of these mice contain an internal ribosome entry site monomeric red fluorescent protein (IRES-mRFP) cassette within exon 2 of the *Id2* gene. In these mice, RFP was highly expressed in all ILC subsets and in splenic and liver NK cells (67). In the current model of ILC development, Lin^−^CD117^+^CD135^−^${\text{α}}_{4}{\text{β}}_{7}^{+}$CD25^−^ ILC progenitors (ILCP) are considered the earliest precursor population giving rise to ILCs downstream of CLPs (68, 69). Analyses of immune cell reconstitution 5 weeks after adoptive transfer of bone marrow-derived Id2^RFP^ ILCPs into sub-lethally irradiated immunodeficient mice showed that all ILC subsets as well as conventional NK cells were present in these mice. Additionally, when Id2^RFP^ ILCPs were sorted and cultured on stroma with cytokines, single-cell cultures gave rise to both single and mixed colonies of ILC1s, ILC2s, ILC3s, and NK cells. To assess PLZF as a distinguishing factor of ILC progenitors, Id2^RFP^ mice were crossed with *Zbtb16*^GFPcre^ mice to generate double-reporter mice. *Zbtb16* is the gene encoding PLZF. *Id2*^+^*Zbtb16*^−^ and *Id2*^+^*Zbtb16*^+^ ILCPs were purified from double-reporter mice and adoptively transferred into immunodeficient mice. Both populations gave rise exclusively to ILC subsets and NK cells with no detection of B cells, T cells or myeloid cells. Sorted *Id2*^+^*Zbtb16*^+^ ILCPs could also give rise to all ILC subsets as well as NK cells in single-cell cultures. Results from these experiments performed with a more sensitive reporter system suggest that conventional NK cells and ILCs are derived from a common early precursor and that neither Id2 nor PLZF distinguishes progenitors with differing lineage potential (67).

Not surprisingly, human ILC development is less well-characterized. A lineage-committed CD34^+^ ILC3 precursor expressing the transcription factor RORγt has been found in tonsil and intestinal lamina propria tissues but not in the peripheral blood, bone marrow or thymus (70). Freud and colleagues also identified a Lin^−^CD34^+^CD45RA^+^CD117^+^IL-1R1^+^RORγt^+^ progenitor population that expressed *ID2* and could differentiate into all ILC types, including conventional CD56^bright^ NK cells, *in vitro*. This progenitor was found in several different secondary lymphoid tissues (SLT) but not in hematopoietic tissues or thymus. Intriguingly, *RORC1* and *RORC2* (encoding RORγ) transcripts were present in all mature ILC subsets and CD56^bright^ NK cells but not CD56^dim^ NK cells (71). This finding contrasts with fate-mapping studies in mice where RORγt expression was found to be restricted to ILCs, and a RORγt^+^ progenitor gave rise to subsets of ILCs but not NK cells (72, 73). The observation of *RORC2* expression in CD56^bright^ but not CD56^dim^ NK cells raises questions about the developmental relationship between these two subsets. It's possible that *RORC2* expression is downregulated during the presumed developmental transition of CD56^bright^ NK cells into CD56^dim^ NK cells. Alternatively, *RORC2* expression could be a lineage-defining factor that marks two distinct lineages (71).

While the two studies referenced above describe ILCs located in SLT, a recent report has extensively characterized human ILCPs that circulate in peripheral blood. These cells are found at a low frequency in blood and are CD45^+^CD7^−^CD56^−^CD25^+^CD127^+^CD117^+^IL1R1^+^CD69^−^. Analysis of progeny from single ILCP cell cultures showed that all ILC subsets as well as NK cells developed from ILCPs. ILCPs as defined in this study represented a heterogeneous population comprised of unipotent and multipotent progenitors, and some ILCPs exhibited the potential to generate both NK cells and ILCs at the single-cell level. In addition to peripheral blood, human ILCPs were identified in cord blood, SLT, fetal liver, and adult lung. Results from this work support the idea that circulating ILCPs can seed various tissues, and that environmental factors within the tissue can “instruct” further differentiation toward the ILC1, ILC2, ILC2, and NK lineages. Some of this instruction is likely given by the presence or absence of Notch ligands and the cytokine milieu (74). Collectively, these studies provide strong evidence that a precursor population exists in humans that has the potential to differentiate into ILCs and conventional NK cells. To what extent the total NK cell pool in humans is derived from an ILC/NK-restricted precursor is unknown and is a challenging question to address. It is possible that many tissue-restricted NK cell populations could arise from an ILC/NK-restricted precursor, while circulating peripheral blood NK cells arise from other CLP, CMP, or GMP populations. Because fate-mapping experiments cannot be carried out in humans for obvious ethical reasons, the continued refinement of humanized mouse models for analysis of human NK cell development might be the best approach for advancing our understanding.

## Adaptive NK Cell Development

Over the past decade there has been considerable interest in the concept of NK cell memory. The idea that NK cells may possess attributes of immunological memory began with the discovery that mouse cytomegalovirus (MCMV) encodes an MHC-like protein (m157) that engages the activating receptor Ly49H on NK cells. This interaction was shown to be important for host protection against the virus (75, 76). Further analysis of the Ly49H^+^ NK cell population in MCMV-infected mice revealed that these cells expanded robustly in the liver and spleen after infection. Following a contraction phase, the remaining Ly49H^+^ cells remained in lymphoid and non-lymphoid organs for several months. Adoptive transfer experiments showed that these “memory” cells could undergo secondary expansion in response to viral challenge and conferred protective immunity (77). An analogous population of NK cells expressing the activating receptor NKG2C expands specifically in response to HCMV (78). While the Ly49H/m157 interaction is crucial for host protection against the virus, the same is not true for the NKG2C/HLA-E interaction. Approximately 4% of humans carry a homozygous deletion of *KLRC2*, the gene that encodes NKG2C. Because of built-in redundancy in the human response to HCMV, NK cells from NKG2C^−/−^ individuals can still mount a response against the virus through other activating receptors (78, 79). This redundancy is reflected in the epigenetically regulated diversification of NK cell signaling and function that has been reported in HCMV seropositive individuals (62, 80). Another more general form of NK cell memory for haptens or viruses has also been described in mice. These NK cells are hepatic and express the chemokine receptor CXCR6 (81). This work has been extended to humans where it has been shown that a population of NK cells expressing tissue residency markers (CD69, CD62L, CXCR6) exhibit recall responses to varicella-zoster virus (VZV) and appear to be very long lived (82).

Little is known regarding the developmental origin or adaptive or memory NK cells. There is circumstantial evidence to suggest that the liver may be a site for NK cell memory acquisition. Two recent studies have characterized liver-resident NK cells from biopsied human tissue. These cells express the liver-specific adhesion molecules CXCR6 and CD49a. High frequencies of these cells also express NKG2C and KIR (83, 84). It is possible that the liver is the primary extramedullary site for the development of adaptive NK cells. These cells could then traffic to sites of infection, expand upon activation and traffic through peripheral blood.

The developmental path from CD34^+^ hematopoietic progenitor cell to adaptive NK cell has not yet been elucidated. It may be that the same lineage-restricted Lin^−^CD34^+^CD38^+^CD123^−^CD45RA^+^CD7^+^CD10^+^CD127^−^ NK cell precursor that has previous been described (33) can differentiate into adaptive NK cells under supportive conditions. Alternatively, there could exist a unique precursor cell that gives rise to an adaptive NK cell lineage. New experimental systems and approaches will likely be needed to understand the ontological relationship between adaptive and canonical NK cells. Currently, there are no studies that have reported on the ability to take canonical NK cells from HCMV seronegative donors and induce and adaptive NK cell state *ex vivo*.

## Discussion

**Figure 1:**
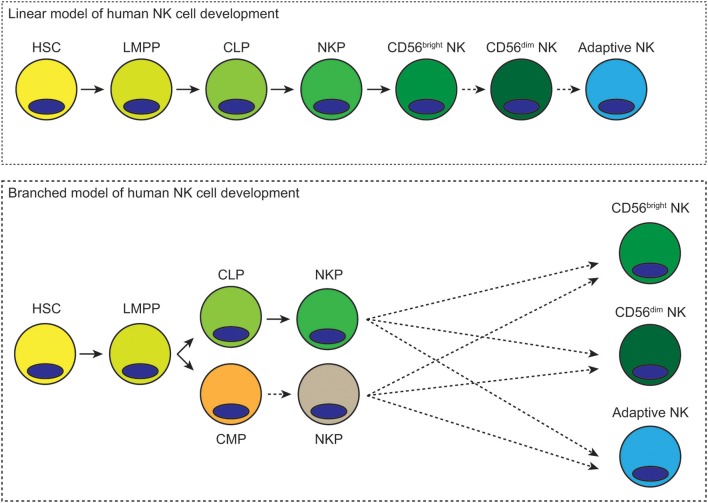
Possible linear and branched models of human NK cell development. In the linear model of human NK cell development, hematopoietic stem cells differentiate into lymphoid-primed multipotential progenitors, which then become common lymphoid progenitors. Lineage commitment occurs at the NK precursor stage. These cells then mature first into CD56^bright^ NK cells and then CD56^dim^ NK cells. Differentiation into adaptive NK cells could subsequently occur in response to viral infection. In the branched model of human NK cell development, hematopoietic stem cells differentiate into lymphoid-primed multipotent progenitors, which then differentiate toward common lymphoid or myeloid progenitors. Either of these progenitors could give rise to NK cell progenitors. These NK cell progenitors could then differentiate into CD56^bright^, CD56^dim^, or adaptive NK cells. Dashed arrows represent hypothetical routes of development/differentiation.

Our understanding of hematopoiesis in general and NK cell development in particular has advanced considerably over the past several decades. There is now increased awareness of the plasticity of hematopoietic progenitor cells and their capacity for differentiating toward multiple lineages. One major unresolved question is whether human NK cells arise from a distinct set of clonal precursors or whether they arise from multi-potent progenitors that also split off into the T cell, B cell or myeloid lineages. If NK cells have a particularly unique ontogeny, at what stage of hematopoiesis do they diverge? Another major question that remains to be resolved is whether CD56^bright^ NK cells represent a distinct lineage or whether they are precursors of CD56^dim^ NK cells. A third major question is the developmental origin of adaptive NK cells and whether they represent a lineage distinct from canonical NK cells. To what degree is NK cell development a linear path from hematopoietic stem cell to terminally mature NK cell, and to what degree is it a branched process where different progenitor cell populations give rise to distinct NK cell lineages (Figure 1)? More in depth investigation and the development of new approaches and technologies should shed more light on these difficult questions and provide more definitive answers. It is also important to keep in mind the myriad differences between mice and humans with regards to hematopoiesis and immune cell development.

While these questions are interesting from an academic perspective, advancements in our understanding of human NK cell development will be critical for the development of new immunotherapies. One major challenge is how to successfully treat patients with solid tumors with an NK cell therapy. We know that NK cells exist within peripheral tissues where tumors can arise and can infiltrate the tumor microenvironment. However, we do not know the precise developmental pathway these NK cells take, which precursors they differentiate from or what environmental cues instruct their maturation. With this knowledge, we could potentially guide the differentiation of either a subset of CD34^+^ progenitors or induced pluripotent stem cells (iPSCs) *in vitro* to generate NK cells for adoptive immunotherapy that will home to specific tissues and persist. Similarly, we know that certain subsets of NK cells respond specifically to HCMV, VSV, and EBV infections. There is potential to develop an NK cell-based immunotherapy to treat patients who have complications from these infections. For this approach to become a reality, we need a better understanding of the developmental origins of these virus-specific NK cell subsets.

## Author Contributions

FC, BG, and JM wrote the manuscript.

### Conflict of Interest Statement

The authors declare that the research was conducted in the absence of any commercial or financial relationships that could be construed as a potential conflict of interest. The reviewer SS and handling editor declared their shared affiliation at the time of review.
